# Benefits of Motor Imagery for Human Space Flight: A Brief Review of Current Knowledge and Future Applications

**DOI:** 10.3389/fphys.2019.00396

**Published:** 2019-04-11

**Authors:** Aymeric Guillot, Ursula Debarnot

**Affiliations:** ^1^Inter-University Laboratory of Human Movement Biology-EA 7424, University of Lyon, University Claude Bernard Lyon 1, Villeurbanne, France; ^2^Institut Universitaire de France, Paris, France

**Keywords:** mental practice, weightlessness, motor performance, mental processes, microgravity

## Abstract

Motor imagery (MI) is arguably one of the most remarkable capacities of the human mind. There is now strong experimental evidence that MI contributes to substantial improvements in motor learning and performance. The therapeutic benefits of MI in promoting motor recovery among patients with motor impairments have also been reported. Despite promising theoretical and experimental findings, the utility of MI in adapting to unusual conditions, such as weightlessness during space flight, has received far less attention. In this review, we consider how, why, where, and when MI might be used by astronauts, and further evaluate the optimum MI content. Practically, we suggest that MI might be performed before, during, and after exposure to microgravity, respectively, to prepare for the rapid changes in gravitational forces after launch and to reduce the adverse effects of weightlessness exposition. Moreover, MI has potential role in facilitating re-adaptation when returning to Earth after long exposure to microgravity. Suggestions for further research include a focus on the multi-sensory aspects of MI, the requirement to use temporal characteristics as a measurement tool, and to account for the knowledge-base or metacognitive processes underlying optimal MI implementation.

## Introduction

One main unique aspect of spaceflight is that astronauts do not feel the effects of gravity and therefore experience a weightlessness sensation, also called zero gravity, or microgravity. Technically, escaping the bonds of gravity, which can be simulated in parabolic flights, is known to disrupt both vestibular and proprioceptive systems with symptoms including confusion in the sense of up and down affecting the body schema (Grabherr et al., 2007), the body orientation (Massion et al., 1998; Lackner and Dizio, 2000), and motor control (Papaxanthis et al., 1998; Lackner and Dizio, 2000). Furthermore, additional long-term consequences of extended missions in space include bones weakening (osteoporosis), loss of muscle mass, strength, and endurance (Fitts et al., 2000; Williams et al., 2009), and decrease of blood volume and immunodeficiency (White and Averner, 2001; Williams et al., 2009). Neural studies further revealed changes in the patterns of brain activation after long missions in space (Van Ombergen et al., 2017). For instance, Roberts et al. (2017) reported narrowing of the central sulcus and the cerebrospinal fluid spaces at the vertex, in addition to an upward shift of the brain, which may cause visual impairment and intracranial pressure syndrome. Interestingly, when asking astronauts to either perform pure motor imagery (MI) or visuospatial imagery, Demertzi et al. (2016) observed greater activation of the supplementary motor area post-flight during MI. These results provide evidence that exposure to microgravity might not only affect the human physiology but also the human brain.

Research comparing motor performance in normogravity and microgravity contexts has accumulated in the past 25 years (for a recent review, see Macaluso et al., 2018). Divergent findings have emerged with some studies reporting alteration of movement accuracy and control (Bock et al., 1992; Papaxanthis et al., 1998; Bringoux et al., 2012), in addition to movement speed (Carriot et al., 2004; Crevecoeur et al., 2010). On the other hand, others studies failed to find significant differences in motor control and movement patterns (Papaxanthis et al., 2005; Bringoux et al., 2012). In a recent study, Macaluso et al. (2018) provided evidence that humans might be able to maintain the performance of functional goal-directed actions in weightlessness by successfully managing the spatiotemporal constraints of the movement through postural strategies reducing the displacement of the center of mass.

In sum and with the caveat that the findings have been inconsistent, the detrimental effects of microgravity on human sensorimotor skills must be taken into account both before and during the exposure to a weightlessness condition. In order to counteract these effects, astronauts are usually subjected to intense preparation including practice in simulators, training under water, and parabolic flights (Loehr et al., 2015; Kalicinski et al., 2017), and further have an allocated physical exercise program during their mission in space (e.g., Petersen et al., 2016). Active body mobilization remains, however, limited in space whereby astronauts are confronted with a shortage of time to complete such programs. A cost-effective, and non-invasive adjunct to complement physical training to both prepare the astronauts before a spaceflight and compensate for the detrimental effects of weightlessness exposure is MI. MI has demonstrated to enhance physical practice both in terrestrial (Schuster et al., 2011) and astronaut populations (Papaxanthis et al., 2003; Chabeauti et al., 2012; Bock et al., 2015). Finally, there is a paucity of research investigating the effect of MI when returning to normogravity while a strong theoretical basis would support ergogenic effects.

## The Multifaceted Nature of Motor Imagery

Motor imagery is a dynamic mental state during which the representation of a movement is rehearsed without engaging in the corresponding overt execution (Jeannerod, 1994). MI is a multimodal construct which consists of either recalling previously perceived images or envisaging forthcoming events through different sensory modalities. MI has multiple applications in both sport sciences and physical rehabilitation, and there is now converging evidence that MI enhances motor learning (Driskell et al., 1994; Munzert and Zentgraf, 2009; Schuster et al., 2011) and promotes motor recovery (de Vries and Mulder, 2007; Malouin et al., 2013). Interestingly, MI has been shown to improve motor performance both through *online* learning processes, since they occur as a *direct* consequence of practice, and *offline* learning processes (delayed performance improvement), which *indirectly* result from practice (for an extensive review, see Di Rienzo et al., 2016).

Understanding the neural correlates of goal-directed actions, whether executed or imagined, as well as the functional neuroanatomical networks associated with expertise in MI, has been an important achievement in cognitive brain research since the advent of neuroimaging techniques. Accumulated experimental evidences suggest that movement execution and MI share substantial overlap (albeit incomplete) of active brain regions (e.g., Jeannerod, 1994; Munzert and Zentgraf, 2009; Guillot et al., 2012a; Hétu et al., 2013; Hardwick et al., 2018), hence highlighting the functional equivalence between these two forms of practice. The principle of functional equivalence suggests that “motor imagery … should involve, in the subject’s motor brain, neural mechanisms similar to those operating during the real action” (Jeannerod, 2001, pp. S103–S104). Executed movements and simulation (i.e., MI) of the corresponding action engage comparable patterns of connectivity between cortical motor regions (Gao et al., 2011). MI therefore represents an efficient method to stimulate brain motor networks mediating skill acquisition and consolidation (Di Rienzo et al., 2016). Covert and overt practice of the corresponding movement share other similarities. Firstly, the time course of mentally simulated actions has been found to be highly correlated with that of the executed movement (e.g., Decety et al., 1989; Papaxanthis et al., 2002). Certain systematic distortions occur in this temporal relationship influenced by several external factors including action complexity and duration (for reviews, see Guillot and Collet, 2005; Guillot et al., 2012b). Secondly, the peripheral activity of the autonomic nervous system shows similar responses prior and during both MI and actual practices (for review, see Collet et al., 2013). Finally, MI has also been shown to be influenced by biomechanical and motor constraints (Munzert and Zentgraf, 2009). Taken together, these similarities between actual and imagined movements promote MI as a relevant alternative and/or complementary approach to physical practice.

Few studies to date have specifically investigated the specific relationship between MI and microgravity (for review, see Grabherr and Mast, 2010; Table 1). One exception by Papaxanthis et al. (2003) showed that cosmonauts performed and imagined movements with similar durations before and after exposure to microgravity. Interestingly, both MI and actual times were longer 2 days after return to Earth, and returned to pre-flight values 6 days after landing. Their findings strongly support that MI process replicates the neural modifications occurring during the re-adaptation of the motor system on Earth’s gravito-inertial environment. Based on these findings, MI is therefore predicted to accurately mimic motor execution in the microgravity context. Consequently, MI should ideally be performed before, during, and after exposure to microgravity, respectively, to prepare for the sudden lack of gravity after launch, reduce the adverse effects of weightlessness exposition, and facilitate re-adaptation when returning from long exposure to microgravity.

**Table 1:** Previous studies considering the effects of microgravity or zero gravity on motor imagery.

Authors	Type of paper	Main results
Chabeauti et al., 2012	Experimental	Actual durations are significantly longer than motor imagery durations in a weightlessness condition, with imagined durations being similar in normo- and microgravity. Changes elicited by microgravity might therefore hinder the updating of the internal models of action.
Bock et al., 2015	Review/theoretical	Theoretical guidelines of motor imagery training pograms designed to reach an optimal level of preparation before exposure to microgravity, and improve performance of astronauts upon return to Earth, before landing.
Grabherr and Mast, 2010	Review/theoretical	By considering the effects of microgravity on the ability to perform mental and motor imagery, the authors highlighted the lack of research investigating the effects of weightlessness on imagined movements, in particular during exposure to microgravity.
Kalicinski et al., 2017	Review/theoretical	Motor imagery of actions which are impossible on Earth (full body floating task) remains possible - although being degraded - and might thus be beneficial for preparing astronauts before their missions and space flights.
Papaxanthis et al., 2003	Experimental	Actual and motor imagery durations were strictly similar both before and after exposure to microgravity. Interestingly, these durations likewise increased 2 days after return to Earth, before returning to approximate pre-flight values 6 days after landing.


### Performing Motor Imagery Before Microgravity

As earlier outlined by Bock et al. (2015), MI should be performed before exposure to microgravity, for at least three main reasons: (i) enhancing the ability to perform MI and the quality of the MI experience, (ii) preparing for exposure to the weightlessness condition, and specifically prepare astronauts for the sudden lack of gravity after launch, and (iii) providing relevant pre-adaptation of MI practice which is likely to be degraded during microgravity exposure.

Preventing the negative effects of microgravity on MI during exposure to microgravity is of particular interest. A study of such detrimental effects was reported by Chabeauti et al. (2012), who provided evidence that actual durations were significantly longer than imagined durations in a weightlessness condition, and that imagined durations did not differ when comparing data collected in normogravity and microgravity. These results suggest that changes elicited by microgravity are likely to hinder the updating of the internal models of action, hence altering the ability to preserve the temporal congruence between actual and MI performance. Based on these findings, developing MI *before* exposure to microgravity, and notably the ability to decrease MI speed, might contribute to preserve the internal models of action, and therefore promote the ability to preserve the temporal equivalence between MI and physical practice during the subsequent flight. In particular, performing slow-motion imagery is known to facilitate a more in-depth and detailed analysis of motor skills being imaged (O and Hall, 2013), which may be useful when anticipating the effects of microgravity on actual performance speed.

While not directly reflecting the influence of microgravity *per se*, Kalicinski et al. (2017) recently designed a study investigating the ability to imagine a movement which is not possible to perform under the presence of gravity (i.e., in a floating position). Although MI remained possible, they found that the elaboration and the control of mental images were degraded, and therefore postulate that MI of vestibular challenging movements might be relevant for astronauts, during their pre-flight training. Specific accurate MI exercises might thus be designed with a focus on the forthcoming lack of gravity. In this particular situation, external visual imagery, which requires to be dissociated from the action itself, might be particularly relevant. Concurrently, developing the ability to imagine the movement mainly from a visual perspective, i.e., without integrating the feeling of the sensations and balance elicited by the action during kinesthetic imagery, may contribute to prepare astronauts for exposure to microgravity.

### Motor Imagery to Reduce the Adverse Effects of Microgravity During the Flight

As mentioned previously, converging evidence supports the contention that MI improves motor performance and facilitates motor learning in a similar way (i.e., functionally equivalent) to actual practice of the corresponding movement. Neuroimaging studies provided evidence that the cerebral plasticity occurring during the incremental acquisition of a motor sequence through actual practice was also reflected during MI (Lafleur et al., 2002; Jackson et al., 2003). In a seminal study, Pascual-Leone et al. (1995) reported an enlargement of the cortical representation of target muscles controlling a motor sequence learnt by MI, thus providing clear evidence of neuroplasticity from MI practice. Interestingly, in recent years, researchers investigated how optimally combining embedded MI and physical practice of the same movement in order to achieve peak performance. Allami et al. (2014) provided evidence that MI may replace up to 75% of the physical training if a minimal ratio of physical practice is delivered. Similarly, Reiser et al. (2011) reported strength gains after different ratios of MI and physical practice. In clinical settings, Malouin et al. (2004) observed that one session of rehabilitation including 15% of MI and 85% of physical practice resulted in comparable motor performance gains to 3 weeks of physical therapy. The same authors reported that prior MI practice might reduce by four the amount of physical practice required to reach the same level of performance (Malouin et al., 2009). Taken together, these findings emphasize the importance of embedding MI during physical practice training programs. MI is particularly useful when this physical practice training is restricted, for example, during spaceflights. As suggested by Kalicinski et al. (2017), MI exercises during space flight should also be performed with a focus on adjusting to gravitational forces to prepare astronauts for daily activities after landing. While MI must be seen as a complement to physical practice, rather than being an alternative, MI may need to be the predominant form of training at certain times during long flights, when there is limited space for exercise equipment.

Another important reason to consider the use of MI in weightlessness conditions is its expected beneficial effects on the limitation of strength loss. There is a general consensus that MI contributes to improve strength (Yue and Cole, 1992; Ranganathan et al., 2004; Yao et al., 2013), muscle activation and force performance (Di Rienzo et al., 2015; Grosprêtre et al., 2017). More importantly, MI has been shown to limit the loss of strength in patients with motor disorders and persons suffering from immobilization (Newsom et al., 2003; Lebon et al., 2012; Clark et al., 2014). As physical exercise and active mobilization are limited when facing weightlessness conditions, MI appears to be a plausible alternative to physical practice which may compensate for the lack of actual muscle contractions, which are known to affect the sensorimotor representations of the immobilized body parts (Meugnot et al., 2014). Specifically, the slowdown of the sensorimotor processes may be counteracted by kinesthetic imagery practice, while these beneficial effects would not systematically appear with visual imagery (Meugnot et al., 2015).

Overall, it is important to keep in mind that the nature and the quality of MI (i.e., the ability to preserve the temporal equivalence between imagined and actual times) during exposure to microgravity should be thoroughly controlled as MI is likely to be degraded in weightlessness conditions. Assessing and developing the individual MI ability before the mission therefore appears another critical prerequisite to maintain its accuracy during the flight.

### Performing Motor Imagery After Microgravity

To our knowledge, no study has investigated the selective effects of MI after exposure to microgravity in order to specifically determine whether it may facilitate re-adaptation to normogravity. Experimental studies including MI trials after microgravity were mainly designed to compare with data collected before spaceflights. Interestingly, Papaxanthis et al. (2003) showed that on the second day post-flight, both actual and MI durations increased compared to pre-flight measurements, before returning to approximate pre-flight values 6 days after landing. Data therefore revealed similar evolutions for both types of practice, hence highlighting that dynamics of the motor system are appropriately reflected during MI.

Practically, astronauts exhibit pronounced long-term microgravity-related effects requiring weeks to months of rehabilitation for complete recovery. As MI has been shown to promote recovery and functional rehabilitation in patients with motor disorders (Malouin et al., 2013), specific MI exercises may be performed to facilitate re-adaptation and therefore limit the harmful consequences of long exposure to microgravity. Based on findings by Papaxanthis et al. (2003) and predictions derived from simulation theory (Jeannerod, 2006), MI would be expected to have a priming effect on expected physical changes when returning from a weightlessness period. Practically, astronauts spend weeks engaged in hypertrophy training to rebuild muscle and repairing bone after a long mission. Post-flight MI exercises might thus be practiced to promote strength (re)gains and facilitate fluid and effective movement execution of complex motor and balance tasks.

## Conclusion: How to Implement MI Into the Preparation and Mission of the Astronauts

Motor imagery should ideally be performed before, during, and after exposure to microgravity to prepare for the lack of gravity, counteract the effects of weightlessness and promote the re-adaptation to normogravity. A quite similar theoretical viewpoint had been nicely proposed by Bock et al. (2015), who more specifically focused on the preparation period few days before landing. These authors developed two phases of individual MI training program to reach an optimal level of preparation before exposure to microgravity. In the first phase, astronauts should familiarize with MI and develop their MI ability, concurrently with physical practice. Practically, programs might incorporate MI of exercises related to conditions encountered during the forthcoming flight. The second step would be scheduled a few days just before landing and improve performance of astronauts upon return to Earth. Whereby MI might be used and provide before landing and improve performance of astronauts upon return to Earth benefits such as during and after the flight should certainly be extended at other times.

MI is a multimodal construct and should ideally combine the different imagery modalities, including visual imagery through the first and third-person perspectives, as well as kinesthetic imagery. As mentioned previously, this latter form of imagery practice, which requires to feel sensations usually elicited by the action, including force and balance, may be of particular interest during and after the flight, while external visual imagery may be more relevant before the flight. There is further converging evidence that including kinesthetic imagery into MI programs specifically contributes to enhance motor performance and limit strength loss. These benefits may thus be of particular interest to further limit strength loss during the flight and promote strength (re)gains after the flight. Another critical issue relates to the timing of mentally simulated movements. As the ability to achieve temporal congruence between imagined and actual practice is likely to be altered during exposure to microgravity, it is important to develop such capacity before the launch, and to carefully control it while practicing during the flight. Based on data reported by Chabeauti et al. (2012), voluntarily modulating MI speed may therefore be punctually relevant, in order to compensate for the time distortion induced by zero gravity and the corresponding lack of updating of internal models. This remains a working hypothesis awaiting experimental research, as previous data in the field of sport provided strong evidence that voluntarily decreasing imagery speed might similarly affect subsequent actual speed. Finally, few experimental studies highlighted the influence of circadian rhythms on MI accuracy, most especially on MI temporal features (Gueugneau et al., 2009, 2017; Gueugneau and Papaxanthis, 2010; Debarnot et al., 2012; Rulleau et al., 2015). Based on these findings providing evidence of harmful effects of time-of-day on accuracy of motor predictions, MI exercises should ideally be performed within the same period of the day. To account for the above dimensions of imagery in an applied context, interventions would need to include specific training on the metacognitive aspects of MI. Specifically, knowledge-based training on how to apply MI optimally would support any interventions (MacIntyre et al., 2014). Overall, future experimental studies are certainly needed and encouraged to confirm all expected and theoretical beneficial effects discussed in the present paper. Developing MI ability might be relevant for ongoing space tourism or personal spaceflight projects, which begin to appear for leisure or business purposes.

## Author Contributions

AG and UD participated to the writing of the manuscript and reading to the final version of the manuscript.

## Conflict of Interest Statement

The authors declare that the research was conducted in the absence of any commercial or financial relationships that could be construed as a potential conflict of interest.
